# Methane Single Cell Protein: Potential to Secure a Global Protein Supply Against Catastrophic Food Shocks

**DOI:** 10.3389/fbioe.2022.906704

**Published:** 2022-07-25

**Authors:** Juan B. García Martínez, Joshua M. Pearce, James Throup, Jacob Cates, Maximilian Lackner, David C. Denkenberger

**Affiliations:** ^1^ Alliance to Feed the Earth in Disasters (ALLFED), Fairbanks, AK, United States; ^2^ Department of Electrical and Computer Engineering, Western University, London, ON, Canada; ^3^ FH Technikum Wien, Wien, Austria; ^4^ Circe Biotechnologie GmbH, Wien, Austria; ^5^ University of Alaska Fairbanks (Mechanical Engineering and Alaska Center for Energy and Power), Fairbanks, AK, United States

**Keywords:** global catastrophic risk, existential risk, single cell protein, methanotrophic bacteria, resilient food, food security, global catastrophic food shock, nuclear winter

## Abstract

Global catastrophes such as a supervolcanic eruption, asteroid impact, or nuclear winter could cause global agricultural collapse due to reduced sunlight reaching the Earth’s surface. The human civilization’s food production system is unprepared to respond to such events, but methane single cell protein (SCP) could be a key part of the solution. Current preparedness centers around food stockpiling, an excessively expensive solution given that an abrupt sunlight reduction scenario (ASRS) could hamper conventional agriculture for 5–10 years. Instead, it is more cost-effective to consider resilient food production techniques requiring little to no sunlight. This study analyses the potential of SCP produced from methane (natural gas and biogas) as a resilient food source for global catastrophic food shocks from ASRS. The following are quantified: global production potential of methane SCP, capital costs, material and energy requirements, ramp-up rates, and retail prices. In addition, potential bottlenecks for fast deployment are considered. While providing a more valuable, protein-rich product than its alternatives, the production capacity could be slower to ramp up. Based on 24/7 construction of facilities, 7%–11% of the global protein requirements could be fulfilled at the end of the first year. Despite significant remaining uncertainties, methane SCP shows significant potential to prevent global protein starvation during an ASRS at an affordable price—US$3–5/kg dry.

## Highlights

● Methane single cell protein has significant potential as resilient food for catastrophes.
● Protein requirements for the entire global population could be fulfilled in 2.5–4.5 years.
● Stranded natural gas (vented, flared, or re-injected) could cover most of the requirements.
● The product would be affordable at an expected retail cost between US$3–5/kg dry.
● Several recommendations and interventions were highlighted to expedite the crisis response.

## 1 Introduction

The risk that a food shock will reduce global food production by about 10% has been estimated to be ∼80% within this century (Bailey et al., 2015), and the chance of a near-total food production loss could be as high as 10% (Denkenberger and Pearce, 2014; Denkenberger and Pearce, 2015). As the COVID-19 pandemic has shown, even disasters not directly related to the food system can substantially increase the population at risk of starvation (Ahn and Norwood, 2020; Laborde et al., 2020), and affect other major systems such as energy (Brosemer et al., 2020), which are integral to the food system. Such events are most dire for countries critically dependent on food imports (Manero et al., 2020; Manheim and Denkenberger, 2020; Shokrani et al., 2020). A scenario on the order of a magnitude of 10% food production loss, such as severe pollinator loss or abrupt climate change, could still result in mass starvation (Beasley, 2020). While “incremental” climate change from global warming over many decades is often considered in the food security literature, there has been limited discussion of more abrupt or extreme climate changes, which have the potential to create severe, sudden food shocks (Denkenberger and Pearce, 2015) with very short windows for adaptation.

There is a clear need for more work on preparedness. Several mechanisms exist for the occurrence of an abrupt global food shock due to agriculture’s dependency on stable environmental conditions such as sunlight, temperature, and precipitation, as well as the dependency of agricultural yields on functioning supply chains. There is an increasing risk over time of concurrent severe weather events causing a multiple breadbasket failure which would further exacerbate global food insecurity (Pham et al., 2022). However, the most extreme food shock that could threaten humanity in the near future is likely an abrupt sunlight reduction scenario (ASRS), in which a catastrophe causes a significant reduction in the amount of sunlight reaching the Earth’s surface. Potential causes include a “nuclear winter,” in which the atmosphere is clouded by soot from a global nuclear war causing the burning of cities, with triggers less likely including a supervolcanic eruption or asteroid/comet impact (Cirkovic, 2008; Denkenberger and Pearce, 2014). Consequences such as subzero temperatures across the Northern Hemisphere summer (Robock et al., 2007; Coupe et al., 2019) would preclude conventional agriculture for many years (Denkenberger et al., 2017), leading to a global catastrophic food shock (GCFS) from a near-total global agricultural production loss. Such events, which would affect human well-being globally and even imperil modern civilization, are categorized as global catastrophic risks (Bostrom and Cirkovic, 2011). A severe ASRS is addressed in this study as an extreme limiting case scenario, in part because the solutions that would work even under such circumstances could be useful for any scale of catastrophe. A global industry is considered to remain largely functioning after the catastrophe; other GCFS scenarios involve a loss of industrial capacity (Denkenberger et al., 2021), requiring different solutions (Denkenberger et al., 2017). These types of events would demand radical innovation in food production, and a variety of complementary solutions would be required to forestall mass starvation: we named these as resilient food solutions (Pham et al., 2022).

Given that an ASRS could last 5–10 years (i.e., nuclear winter), the cost of storing sufficient amount of food for the global population is estimated to be extremely high in comparison to producing resilient foods that require less or no sunlight (Denkenberger and Pearce, 2015; Denkenberger et al., 2019). For example, in an ASRS, cool-tolerant crops could be relocated to more adequate climates (Pham et al., 2022), simple greenhouses could be built on the tropics (Alvarado et al., 2020), and global seaweed production could be quickly ramped up (Mill et al., 2019), sugar could be produced from lignocellulosic biomass (Throup et al., 2022), synthetic fat could be produced from hydrocarbons (García Martínez et al., 2022), acetic acid could be produced from CO_2_ *via* microbial electrosynthesis (García Martínez et al., 2021a), mushrooms grown on the residues from logging, cellulose-digesting ruminants, and insects could be used as a food source (Denkenberger and Pearce, 2015), and leaf protein concentrates could be obtained (Pearce et al., 2019). This work studies the use of microbial protein produced *via* methanotrophic bacteria as a potential component of a food-crisis response. Resilient foods could be instrumental in avoiding starvation and sustaining society in the face of severe food shocks. Indeed, protein scarcity during wartime sparked the initial interest in microbial protein (Ritala et al., 2017).

Microbial protein, referred to as single-cell protein (SCP), has been proposed as an alternative to animal products in meeting the growing global protein demand (Ritala et al., 2017). Outside of the context of a disaster, it is being considered as a sustainable alternative protein by an expanding body of literature (Claassens et al., 2016; Matassa et al., 2020; Leger et al., 2021; Khoshnevisan et al., 2022). A variety of microorganisms, including bacteria, fungi, and microalgae, can be cultivated for SCP production (Ritala et al., 2017). Fungal SCP is already sold for human consumption under the brand name Quorn; however, the current production process relies on sugar (Ritala et al., 2017), a human-edible feedstock, making this product less useful in a GFCS. Conversely, during an extreme food supply crisis, it could be desirable to produce SCPs for human consumption from non-human edible feedstocks such as hydrocarbons, carbon dioxide (CO_2_), or available biowastes (Matassa et al., 2020), rather than using feedstocks such as sugars which could be more efficiently used for direct human consumption in such a dire situation. This would effectively increase food availability by virtue of obtaining a nutritionally rich product from inedible sources. Additionally, SCPs obtained from these resilient feedstocks would not compete on the input side with traditional protein sources. Methanol can also be used as an ASRS-resilient feedstock for SCP production, as was used for the first commercially available microbial protein product: Pruteen, from Imperial Chemical Industries. However, methanol is more localized and produced in smaller quantities than methane. Hydrocarbons can also serve as an ASRS-resilient feedstock for SCP production (Jenkins, 1988), specifically the paraffin components of petroleum which can also be used to produce synthetic fat (García Martínez et al., 2022).

Much research has focused on SCPs on the grounds of sustainability due to their low water and land use (Matassa et al., 2015; Matassa et al., 2016; Pikaar et al., 2018; Sillman et al., 2019; Matassa et al., 2020). Methane-based bacterial SCP can use orders of magnitude-less water and land than traditional protein sources such as meat and plant-based proteins. For example, *Feedkind* ^*TM*^ approximately uses 100 times less water and 1,000 times less land per ton compared to soybeans (Cumberlege et al., 2016). Compared to other sunlight-independent food sources such as crops grown under artificial light or microalgae grown in photobioreactors, bacterial SCP has very high energy efficiency (Alvarado et al., 2021). Due to its CO_2_ emissions, SCP from natural gas is less sustainable than renewable hydrogen-based bacterial SCP, a similar type of SCP that could also be used as resilient food in GCFS (García Martínez et al., 2021b). However, natural gas SCP’s lower requirements in terms of equipment imply a faster ramp-up and lower cost, which are fundamental characteristics for the potential of methane SCP as resilient food for GCFS (García Martínez et al., 2021b). This is because H_2_ SCP requires the construction of costly hydrogen-production facilities while methane SCP can leverage existing natural gas-extraction facilities. Most significantly for the purpose of crisis response, these types of SCP can produce high-quality, protein-rich food independently of traditional agriculture, specifically avoiding the need for sunlight, and human edible inputs. For these closed-environment food production systems, exposure to several risk factors that affect conventional agriculture is greatly reduced or completely nullified, such as: institutional factors (i.e., trade restrictions), abiotic factors (i.e., environmental degradation, extreme weather events, or climate variability), and biotic factors (i.e., pathogens or pests) (Tzachor et al., 2021).

Methanotrophic bacteria can be found in nature where methane naturally occurs, e.g., in swamps or tundra regions, and have been studied as a food source since the 1970s. Interest in them declined, but has now been recovering thanks to their ability to produce high-quality protein with minimal land and water use. Methane SCP is one of the most advanced and accessible SCP production technologies, and is currently on the verge of large-scale commercialization (Flanagan, 2022). Methane is a relatively ubiquitous resource, and gas fermenters can be built at different scales, with a typical commercial plant size producing 10,000 to 20,000 tons of protein per year. With several reactors being built on one site with the joint use of utilities, economies of scale allow for production capacities in the order of 100,000 SCPs per year (Crumbley and Gonzalez, 2018; Flanagan, 2022).

Bacterial SCP from methane could potentially become an ingredient in a variety of food products, including solid food like bread, pasta, and plant-based meats, as well as in liquid food and drinks, such as plant-based dairy products, protein shakes, or broths (Southey, 2019). Bacterial SCP from methane has a high-protein completeness because its essential amino acid content is similar to, or higher than, the FAO guidelines (Ritala et al., 2017). Its amino acid content is superior to that of soybean meal, and it boasts of higher protein content in general (Pikaar et al., 2018). To date, there are no publicly available studies of bacterial SCP bioavailability in humans, but recent studies in fish point to bacterial SCP having high digestibility (Glencross et al., 2020; Rajesh et al., 2022). Because of the fat content of methane SCP, its reported caloric content is 22 MJ/kg (Unibio Group, 2020a), noticeably higher than the average 17 MJ/kg caloric content of carbohydrates (U.S. Department of Agriculture, 2016). Methane SCP also boasts of a notable micro-nutritional profile, containing significant amounts of B-group vitamins (esp. B1, B2, B3, B7, B8, and B12), minerals (e.g., iron and magnesium) and essential fatty acids (Duchene, 2016; Silverman, 2020).

However, bacterial SCP has a high content of nucleic acids (8%–12%) (Volova and Barashkov, 2010). This could cause health problems, such as gout and kidney stones (Ritala et al., 2017), if bacterial SCP were to be used as a significant food source for animals with long lifespans. Significant SCP consumption is not recommended for humans, unless the nucleic acid content is reduced during processing prior to use. The maximum safe limit of nucleic acid consumption for an adult human is 4 g/day (Adjei et al., 1995), which is equivalent to the amount present in 234 kcal worth of unprocessed bacterial SCP. This amount of unprocessed bacterial SCP in turn amounts to up-to 59% of the daily recommended protein intake for human adults of 60 g/day. Unibio A/S claims to have developed a method to reduce nucleic acids to below 1% (Jorgensen, 2011).

Conversely, fungal SCP has been considered a safe component of the human diet for several years (U.S. Food and Drug Administration, 2002). Fungal SCP has a particularly low nucleic acid content compared to other SCPs. It is sold after post-processing that further reduces the nucleic acid content (Ritala et al., 2017) to below 2% of the dry weight (Marlow Foods Ltd, 2001). If a human adult’s daily protein requirements were to be fulfilled solely using fungal SCP, the total nucleic acid intake would still remain below the daily safe limit. Research has established that there are little to no threats associated with human consumption of fungal SCP, and this protein source is associated with a low incidence of allergic reactions compared to other sources (Finnigan et al., 2019). Although fungal SCP sets a favorable precedent, bacterial SCP by comparison is considerably studied less as a food source for humans, and requires more studies to establish its safety for human consumption. However, according to Solar Foods, the compositional data of their bacterial SCP product “raises no safety nor allergenicity concerns” (Solar Foods, 2021).

Key players currently pioneering the mass production of methane SCP are Calysta Inc., Unibio A/S, Circe Biotechnologie GmbH, and String Bio Pvt Ltd. While these companies generally appear to be focusing on the production of animal feed such as for the aquaculture sector, all four have explicitly shown interest in producing protein directly for human consumption (TEMASEK, 2020; Circe Biotech, 2021; Jensen, 2021; Lee, 2021). Separately, hydrogen-based SCP production companies such as Solar Foods and Air Protein are already developing the technology to produce human food-grade bacterial SCP and have applied for a regulatory approval for human consumption (Solar Foods, 2021), which is a positive sign with regards both to the sustainability of the SCP for the food concept but also for the possibility of consumer safety studies of bacterial SCP in the near future. This work assessed the viability of SCP as a direct human food source, rather than as an animal feedstock because the caloric conversion efficiency of animal products is low, at 3%–31% (Shepon et al., 2016), which is undesirable during a food-shortage scenario in which the intention is to maximize the calories available for human consumption.

Two key metrics were used to globally characterize the potential of methane SCP as a resilient food: 1) how fast production can be ramped up to rapidly counter agricultural losses, and 2) how inexpensive the production cost is, as this determines the affordability for most economically disadvantaged populations. This work also addresses concerns about material constraints raised in previous studies of microbial protein as a resilient food in ASRS (Denkenberger and Pearce, 2014). The scope is akin to a FEL-1 stage (front-end loading) in which the concept is defined and the preliminary budget estimates are produced, but the level of detail is not yet sufficient for construction (Warner, 2019).

The key aim is increasing resilience to a global catastrophic risk, and more generally to existential risks with the potential to eliminate humanity or its future potential (Bostrom, 2013), since a global famine could be considered an existential risk factor which could weaken our defenses to existential risk. In the face of these risks, prevention is insufficient as a defense layer, where response and resilience ought to be engaged as well (Cotton-Barratt et al., 2020), which is the object of this work.

## 2 Methods

Methane SCP production requires three main inputs: 1) methane, which acts as both an electron donor and a carbon source, 2) a nitrogen source, and 3) an oxygen source. Additionally, some minerals are also needed in smaller quantities. Anaerobic methane fermentation (Switzenbaum et al., 1990) has significantly lower yields and is hence not considered.

The chemical reaction used as a reference is the one proposed for *Methylococcus capsulatus* based on the premises proposed by (Villadsen et al., 2011), namely a yield coefficient of methane to cellular biomass of $Y_{CH_{4}/biomass}=0.8\;$ g biomass/g CH_4_ and a biomass degree of reduction $k_{x}=4.20$ , defined as the number of equivalents of available electrons per mol of carbon. The reaction can be expressed in a general form as shown in Eq. 1.

$$CH_{4}+1.453\;O_{2}\;+\;Nitrogen\;source\;\to \;Biomass\;+\;0.479\;CO_{2}+Water$$ (1)

Given ammonia as a nitrogen source for microbial growth, the resulting overall reaction can be expressed as shown in Eq. 2. The formula $CH_{1.8}O_{0.5}N_{0.2}$ stands for a simplified bacterial cell biomass, leaving aside sulfur and other minor components. The biomass is typically around 70% protein and 30% of other compounds (e.g., fats, carbohydrates, ash, etc.).

$$CH_{4}+1.453\;O_{2}+0.104\;NH_{3}\;\to \;0.521\;CH_{1.8}O_{0.5}N_{0.2}\;+\;0.479\;CO_{2}+1.687\;H_{2}O$$ (2)

**FIGURE 1:**
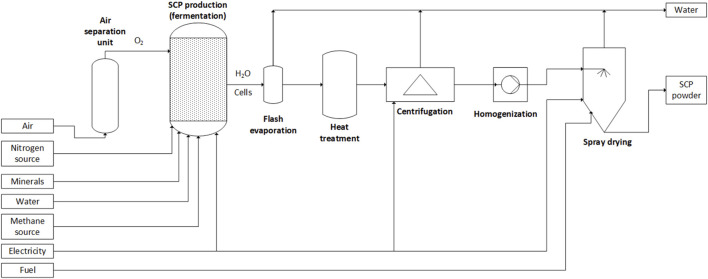
Simplified process flow diagram of the reference methane SCP production process, based on Jorgensen, 2011.

The relevant unit operations, mass and energy flows involved in the proposed reference process are shown in Figure 1. The natural gas and O_2_ are fed together with ammonia and minerals to the bioreactor where the cell growth takes place in a continuous fermentation system, which gives a higher productivity than a batch fermenter. At the outlet of the reactor, the fermentation broth contains an expected biomass concentration of 1%–3% dry weight (i.e., bacterial cells), dissolved gasses, and some unreacted ammonia and minerals. The water is removed *via* mechanical dewatering and drying steps, and the cells are disrupted in the homogenization step to increase digestibility by liberating the nutrients. The final SCP product is obtained in a powdered form, which may be subject to post-processing operations prior to storage. Water recycling, filters, pumps, and heat exchangers are not shown.

A reduced nucleic acid content for the SCP product could be achieved *via* a number of processes, for example, by applying heat treatment (60°C–70°C) to the effluent fermentation broth, or by using alkaline treatment or chemical extraction (Ritala et al., 2017). Significant uncertainty remains as to which nucleic acid removal treatment would be employed in large-scale bacterial SCP production, but heat treatment is shown in Figure 1 in similarity to fungal SCP production. The heat treatment process activates endogenous, RNA-degrading enzymes for short time periods, while controlling the temperature and pH conditions (Anderson and Solomons, 1984). Once degraded, the nucleic acids diffuse out of the cell membrane and enter the liquid fraction, which has been previously separated from the SCP biomass through mechanical dewatering, for example, through centrifugation. It may be possible to remove the need for heat treatments by instead using RNA-degrading enzymes at ambient conditions over longer periods of time, but this process has not been considered in the industrial production studied in this work. Different or no cell disruption techniques may be used instead of homogenization, but the lack of these may reduce digestibility.

The process can use thermophilic bacteria to avoid excessive cooling; see, e.g., (Levett et al., 2016) for a techno-economic assessment of the biopolymer PHB by methanotrophic bacteria. A pure- or mixed-culture operation has been described. The process contains an explosive atmosphere of methane and oxygen, meaning safety precautions have to be taken, particularly in the headspace area. The methane source can be purified upstream of the reactor, or higher hydrocarbons can be consumed by a bacterial consortium.

### 2.1 Methodology Overview

**FIGURE 2:**
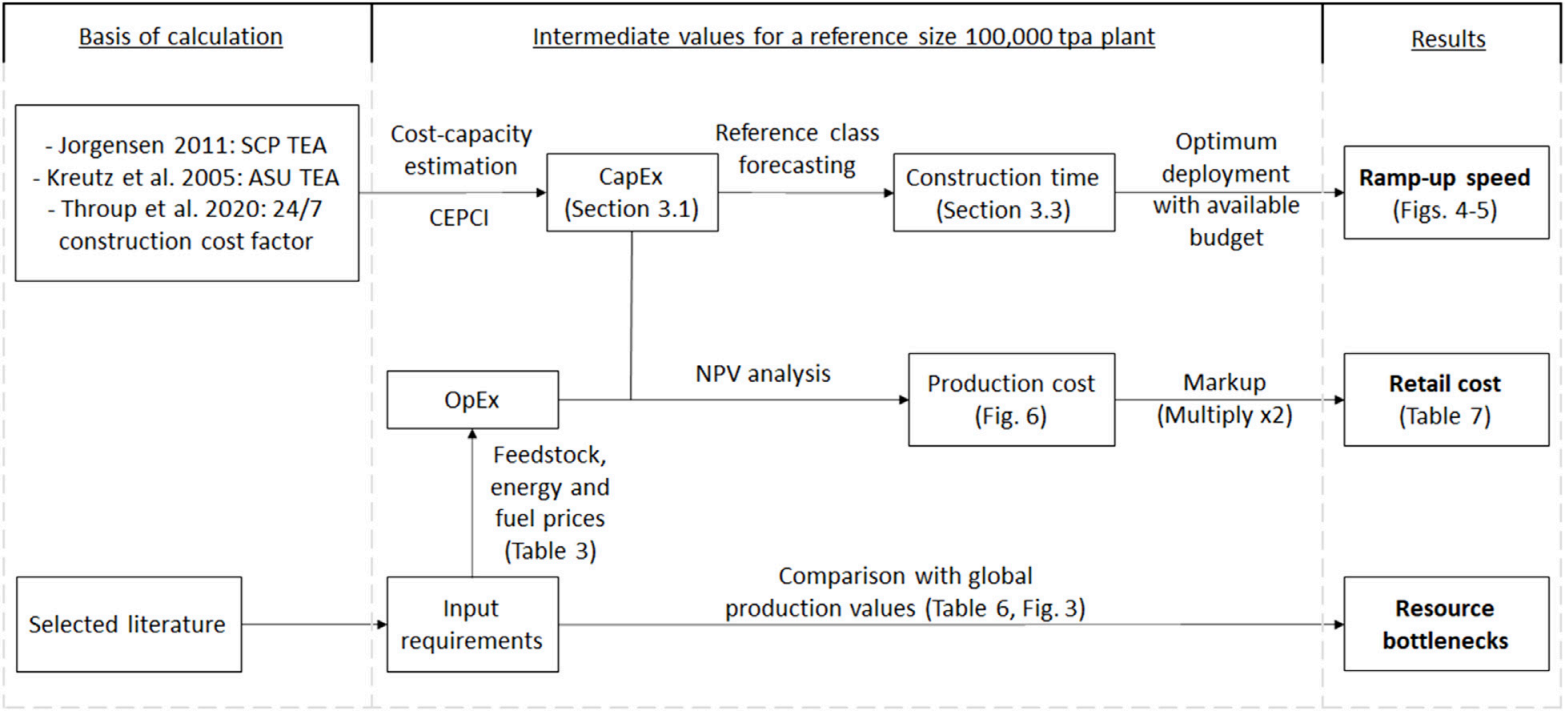
Methodology flowchart (TEA, techno-economic assessment; CAPEX, capital expenditure; OPEX, operating expenditure; NPV, net present value; CEPCI, and chemical engineering plant cost index).

Two key metrics are estimated to characterize the potential of a resilient food source for GCFS: the ramp-up speed (how fast the production can be scaled over time) and the retail price per calorie (how affordable it would be during the catastrophe period). In addition, assessing the global availability of the relevant input resources is key to check for potential bottlenecks to ramp-up fast production. Figure 2 contains an overview of the methodology used to estimate these, which is described in depth in the following sections.

### 2.2 Capital Expenditure Estimation

There is a large uncertainty present in estimating the costs of a large scale “n-th plant” methane SCP production facility because currently, no data on full-scale commercial size plants exist, and “first of a kind” plants are costlier. The fixed capital expenditure (CAPEX) of the SCP plants was based on the data from published industrial estimations by Unibio A/S. For a methane SCP production capacity of approximately 108,000 tons of dry product/year (tpa), the expected capital expenditure is $251 million USD (Jorgensen, 2011). The cost estimate is hereby assumed to represent an n-th plant estimation, meaning that significant cost reductions after the technology reaches a higher level of maturity are not considered in the analysis. This production capacity is representative of that of currently projected full-scale commercial SCP production plants (Lane, 2018; Sefton, 2018; Rosenberry, 2019; Calysta Inc, 2020). After including nucleic acid removal from 9% (Unibio Group, 2020a) to 2%, the installed production capacity of the reference plant becomes 100,800 tpa. Preferably, many (5–10) bioreactors would be present rather than a single massive one for easier construction and operation given this large capacity; compared to the 1,500 m^3^ airlift reactor used to produce Pruteen (Chisti, 1989).

The cost reference does not include the capital cost of O_2_ production. This cost is estimated separately based on the methodology and values proposed by (Kreutz et al., 2005), including the fixed and operating costs. The option of buying O_2_ from industrial suppliers is discarded given the quantities involved in producing enough SCP to feed a significant portion of humanity. The updated capital expenditure of this air separation unit (ASU) is added to that of the Unibio reference factory to obtain the final plant CAPEX.

In an ASRS, food is expected to be scarce after the first few months, as food reserves run out (Denkenberger et al., 2017), making it preferable to increase food production as soon as possible. Fast construction methods are hereby proposed to reduce plant construction time, at the expense of increasing the capital expenditure. The fastest, reasonable cost construction method available is to implement 24/7 construction, reducing the overall construction time to 32% of the original at an increased labor cost of 47% (Throup et al., 2022), according to the methodology and values of Hanna et al., 2007. This value has been conservatively incorporated in terms of a 47% increase in the capital cost of the plant to account for labor constraints. State-of-the-art concurrent engineering could expedite the first steps of factory planning and engineering.

### 2.3 Assessment of Required Resources

Natural gas (NG) is the largest source of methane available to the industry today. One important alternative is biogas obtained from an anaerobic digestion of organic matter, which can be used as a more sustainable alternative methane source (Cumberlege et al., 2016). If the nearly untapped global biogas potential was leveraged in its entirety, it could substitute the equivalent of 26%–37% of the current natural gas production (Jain, 2019). Biogas has been proven as a feedstock for methane SCP production (Jones et al., 2020; Xu et al., 2021a), as well as electrochemically upgraded biogases (Acosta et al., 2020), and artificially synthesized methane from CO_2_ (Xu et al., 2021b).

Different types of natural gas reserves can be defined (Attanasi and Freeman, 2013). Apart from economically exploitable reserves, there are stranded gas reserves, which cannot currently be economically exploited for typical industrial uses. Physically stranded reserves cannot be accessed with our current drilling technology. In contrast, economically stranded reserves are either: 1) too far from their end use to justify transport costs, 2) contained in wells that are too small to justify extraction costs, or 3) associated with oil reserves and thus requiring extraction before the oil can be exploited, but not resulting in profit as a product. The last type is often regarded as an undesirable byproduct of oil extraction and is commonly flared, re-injected, or vented. This associated natural gas is ripe for exploitation *via* methane SCP production because the methane is already being extracted without the need for additional capital cost. This is of considerable value in a GCFS because the resources that would be spent on extraction of further natural gas could instead be spent on the construction of more SCP plants or other resilient foods. In current times, exploiting stranded methane for the production of SCP is estimated to be both economically feasible and more sustainable than the current status quo (El Abbadi et al., 2022). Currently a yearly average of nearly 150 billion cubic meters at standard temperature and pressure (bcm) of natural gas is being flared globally at upstream oil and gas and natural gas-liquefaction plants (Elvidge et al., 2016; World Bank, 2018; EIA, 2019). An estimated yearly average of around 450 bcm of associated natural gas is being re-injected into oil wells (EIA, 2019).

Ammonia is considered as the source of nitrogen due to its widespread global availability from the fertilizer industry; another nitrogen source could be urea. Other potential nitrogen sources include gaseous ammonia recovered from biowaste pyrolysis or gasification (Matassa et al., 2015, 2020), as well as liquid digestate from anaerobic digestion (Khoshnevisan et al., 2019). During an ASRS, global agricultural production would most likely plummet, freeing up ammonia’s production capacity that could be used for SCP production instead. The oxygen is considered to be sourced from cryogenic air separation (Linde process), an industry-standard process from which the majority of industrial oxygen is produced (FMI, 2019), as used by Calysta for methane SCP production (Cumberlege et al., 2016). It is recommended over other air separation processes (e.g., pressure swing adsorption or membrane separation) for a lower production cost in a very large scale oxygen production (UIG, 2006; Alptekin, 2017). Using air directly as an oxygen source may be feasible but could significantly affect key performance parameters such as gas utilization, with a major impact on the efficiency and economics of the process. Modeling and empirical studies, outside the scope of this work, could be required to clarify this. Instead, this work considers the use of pure oxygen as feedstock in line with the practices of major methane SCP companies Calysta and Unibio (Cumberlege et al., 2016; Unibio Group, 2020b).

Resource requirements are summarized in Table 1, and are calculated based on the estimates published for methane SCP, microbial protein requirements more generally, and for chemical industrial equipment. The required utilities to operate a bacterial SCP production plant of a reference size are estimated in terms of electricity and fuel energy requirements. Due to the variability in the properties of natural gas from different locations, a range of variables has been considered for the methane and energy contents of the gas, which will result in a ranged estimation of the natural gas required to fulfill the food requirements. A ranged value of gas utilization has also been considered, which also broadens the overall range. A reactor designed for SCP production would have a gas utilization of at least 80% to be economical, while values of up to 90% have been reported in U-loop reactors (Jorgensen, 2011). The gas utilization affects both the natural gas and O_2_ input requirements of the reference plant. Conservatively, the energy requirement for air separation has been selected as it corresponds to high purity O_2_.

**TABLE 1:** Basis of calculation for the energy requirements of methane SCP production.

Variable	Value	Unit	References
Methane requirement	0.521	mol SCP/mol methane	Villadsen et al. (2011)
Methane content of natural gas	87–98	%mole	Enbridge Gas Inc, (2001)
Gas utilization of reactor	80–90	%	
Energy content of natural gas	35.4–42.8	MJ/m^3^	Engineering ToolBox, (2005)
Energy content of SCP	22	MJ/kg	Unibio Group, (2020a)
Solid content of dryer inlet	20	%	Sillman et al. (2019)
Energy consumption of spray dryer	4880	kJ/kg evaporated water	Baker and McKenzie, (2005)
Electricity to thermal energy usage ratio of spray dryer	1:27	Electricity:thermal	Baker and McKenzie, (2005)
Electricity use of fermentation step	1.6	kWh/kg SCP	Pikaar et al. (2018)
Electricity use of air separation	0.357	kWh/kg O_2_	Aneke and Wang, (2015)

Assessing potential bottlenecks to the ramp-up potential of methane SCP first demands estimating the amount of SCP required to fulfill the food requirements of the global population and the equivalent in terms of the number of reference production plants. The amount of protein and calories available in the SCP product, as well as the requirements for feeding one person, were compared to the number of people globally. Then, the resources required to produce the required amount of SCP are quantified, which includes energy and material resources such as natural gas as the main feedstock, coal for thermal requirements (if adequate), ammonia as a nitrogen source, and electricity to run the process. The decision of whether to use coal or natural gas is not trivial and would depend on the specific location, since using natural gas may be more convenient but would reduce the amount available for SCP production if natural gas was the limiting factor in the location. Some of the natural gas that remains unused after passing through the reactor could be burned to produce energy, but this has been conservatively ignored. Additionally, alternative potential sources of methane are presented for discussion. The values used as a basis for the analysis are summarized in Table 2.

**TABLE 2:** Basis of the calculation for the resource availability analysis.

Variable	Value	Unit	Source
World population	7.8	billion people	United Nations, (2019); Worldometers, (2020)
Recommended protein intake	60	g/person/day	World Health Organization and United Nations University, (2007)
Expected food waste	12	% of calories produced	*
Average daily caloric requirement per person	2,100	kcal/person/day (=1.39 kWh)	World Health Organization, (2004)
Global ammonia production	171	Megaton/year	Research and Markets, (2020)
Global electricity consumption	2,551	GW (1 GWa = 8760, GWh = 8.76 TWh)	Sönnichsen, (2020)
Global coal production	7,337	Megaton/year	Rob Smith, (2018)
Global natural gas production	4,198	bcm/year	IEA, (2019)
Global natural gas flaring and venting	150	bcm/year	Elvidge et al. (2016); World Bank, (2018); EIA, (2019)
Global associated natural gas re-injecting	450	bcm/year	EIA, (2019)
Global biogas production potential	26–37	% of current NG production	Jain, (2019)

*Some amount of food waste throughout the system is unavoidable, regardless of food crisis severity. However, a reasonably low value of food waste, 12%, was considered in the proposed scenario. This value was chosen because food waste is expected to be lower due to increased food scarcity. Moreover, the final bacteria SCP product is a dry product, with a long shelf life, further reducing potential food waste (Denkenberger and Pearce, 2014).

An accurate estimate of the protein content in the final SCP product is essential to estimates of how much of it would be necessary to fulfill the protein requirements of the global population. A protein content range of 50%–80% per kg of dry SCP product resulted from a review of multiple sources (Ravindra, 2000). This range was considered directly when determining, for the required SCP product amounts, the required resource amounts of the four possible energy sources: natural gas, electricity, coal, and ammonia.

### 2.4 Ramp-Up Speed Estimation

We define the ramp-up speed as the increase in food production over time when continuously building as many food production factories as possible with the available resources. In the proposed catastrophe scenario, the ramp-up speed of methane SCP technology during a GCFS would likely be limited by the resources that could be effectively used, including but not limited to: raw materials, energy, qualified labor, and the capacity for equipment construction. We roughly account for these constraints by limiting the budget that can be effectively applied to the 24/7 construction of methane SCP plants to a value of $489 billion per year (Damodaran, 2020), which is the capital expenditure on adjacent industries whose resources could be redirected such as chemicals, power, pulp and paper, utilities, and beverages. It is uncertain if workers of other, more disparate industries could be retrained fast enough to build and operate methane SCP plants. Thus, the average number of facilities that could be constructed in 1 year is obtained by dividing this total yearly CAPEX budget by the cost of a reference size factory.

The time taken to construct a facility is logarithmically related to the cost of the facility. The construction time is estimated by reference class forecasting, using a logarithmic regression model based on the data from previously built factories (Martin et al., 2006). Given the urgency of the scenario, 24/7 construction is assumed to be used, which is estimated to reduce the construction time to 32% of this value (Throup et al., 2022). The number of facilities that could be built per construction “wave” is calculated by dividing the amount of plants that can be built per year by the number of waves per year. For example, the first wave can be seen in the ramp up graphs as the first step increase in food production, shortly followed by another increase representing the moment that the factory transitions from startup production to full production, and later by another increase that represents the second wave.

The startup period is the time of reduced production between mechanical completion and the start of the operation. An average production capacity of 50% applies, and it is considered to last one-fourth of the construction time at regular speed (Humbird et al., 2011). Delays prior to plant construction also affect construction timelines. A time period of 4 weeks is assumed, which is the time it took for complex industries to convert and scale production of relevant supplies during the COVID-19 pandemic (Betti and Heinzmann, 2020). More details on ramp-up speed estimation can be found in Throup et al., 2022 and García Martínez et al., 2021b, including an example of the method on the supplementary material of the latter.

### 2.5 Economic Analysis

A net present value (NPV) analysis was performed by calculating the required revenue for a standard unit of the SCP product when NPV equals zero. This analysis enabled estimates of the break-even cost of the product. To estimate the timeframe of the plant operation, a timeframe of 6 years was used. This timeframe is shorter than those typical of chemical plants, and represents the period in which industrial food production factories could operate during an extreme food shock. This is representative of the duration for a period with little sunlight caused by a nuclear winter. The increased capital cost from the 24/7 construction applies. At the end of the 6 year period, the equipment was considered to be depreciated, corresponding to the time of coldest temperatures. In reality, some lower priced food could be sold for longer, there would be some salvage value, or the systems could be built less expensively (less durably), so this is a conservative assumption. To account for the time value of money, a 10% discount rate was used, consistent with recommendations for economic analyses facing an absence of statistical data for the given technology (Short et al., 1995). For comparison, the same analysis was performed for normal conditions outside of a catastrophe, namely a typical plant lifetime of 20 years and a regular construction cost.

The operating costs are estimated based on the prices from Table 3 and the energy resource requirements for a reference plant from Table 4. For the electricity cost, the typical cost for the aluminum industry was taken to represent the low end of the uncertainty range, and the higher end of the uncertainty range was represented by the current European industry average. Thermal energy costs were calculated based on the cost of the amount of coal required to reach the thermal energy requirements. The total variable operating costs included the electricity and thermal energy costs described previously, plus an additional $10.6 million USD for other operating costs, as well as a further 6.5$ million USD for overheads (Jorgensen, 2011). The working capital was presumed at $32.6 million USD. The financial parameters, with a federal tax rate of 35%, are based on the analysis of (Humbird et al., 2011). Financing sources were taken to comprise 70% equity (with an assumed 10% return on investment) and 30% loaned capital, assuming an interest rate of 8% per annum and a 10-year repayment period.

**TABLE 3:** Energy resource cost ranges considered.

Price range	Low	Middle	High
Electricity price	Global low Burns, (2015)	U.S. average EIA, (2020a)	Europe average Eurostat, (2019)
($/kWh)	0.03	0.07	0.13
Natural gas price	Flared, vented or reinjected NG	10-year average Markets Insider, (2020b)	10-year maximum Markets Insider, (2020b)
($/MWh NG)	0.00	11.26	16.51
Coal price	Global low EIA, (2020b)	Average Markets Insider, (2020a)	10-year high Markets Insider, (2020a)
($/tonne)	11.60	45.80	80.00

**TABLE 4:** Energy requirements of methane SCP production per step in kWh over the dry mass of product.

Step	Energy requirement (kWh/kg SCP)	
	90% gas utilization, low NG energy content	80% gas utilization, high NG energy content
Fermentation	1.6	
Centrifugation	0.8	
Spray drying	5.8	
Air separation	1.5	1.7
Energy equivalent of required natural gas	21.2	32.5
Total energy requirements for methane SCP production	31.0	42.5

## 3 Results

### 3.1 Capital Expenditure Estimation

The updated capital cost of the air separation unit required to fulfill the plant’s O_2_ requirements is estimated at $67 million USD based on Kreutz et al., 2005. Together with the other capital costs the total amounts to $329 million USD for a regular construction, or $481 million for the 24/7 construction. This would be equivalent to a CAPEX per unit of installed capacity of $3,300/tpa and $4,800/tpa, respectively. All costs are updated to 2020 values using the Chemical Engineering Plant Cost Index (CEPCI). These figures are conservatively estimated from the “first of a kind” plant proposed by Unibio; n-th plant facilities are at least 15% cheaper, in the authors’ experience.

### 3.2 Required Resources and Operating Expenditures

The energy requirements estimated for each step are shown in Table 4. All values are calculated from Table 1: the natural gas requirement is obtained based on the proposed reaction stoichiometry, range of methane content, energy content of natural gas, and gas utilization. The air separation energy requirement is calculated based on the energy use of the separation system from the literature, the gas utilization, and the reaction stoichiometry. The fermentation and centrifugation energy requirements are taken from a resource analysis study. Spray-drying energy requirements are obtained from the industry average of a study on industrial spray-drying data and the expected solid content of the inlet stream. The median energy requirements of spray drying were lower, but the average was selected as a more conservative assumption. All values are corrected for nucleic acid removal.

The energy analysis results for a reference plant are shown in Table 5. The values are estimated based on Table 4 and the production capacity. The caloric energy efficiency is estimated as the amount of energy invested in producing the SCP in comparison with its caloric content. The actual energy and efficiency values vary depending on the gas utilization and energy and methane contents of the gas. The estimated methane requirements have been compared against the value proposed by Bio Protein A/S (now part of Calysta). They showed a utilization of 2 m^3^ of methane per kg of SCP produced (Babi and Price, 2010), in accordance with the value obtained here for high gas utilization, high methane content, and no nucleic acid removal, which denotes the conservativeness of the present estimations.

**TABLE 5:** Energy analysis results for a reference plant. The ranges are based on the intervals of gas utilization and energy content of the natural gas input considered.

Variable		Low end	High end	Unit
Total energy requirements of reference plant		359	489	MW
Of which electricity is		48	50	MW
Overall energy efficiency		20.0	14.6	%
Natural gas requirement of SCP production		0.218	0.276	bcm/year
Thermal energy requirements in terms of:	Coal	62,039		ton/year
	Natural gas	0.048	0.058	bcm/year

The share of global resources that would be required to fulfill the protein requirements of the global population *via* methane SCP is shown in Table 6 for both ends of the expected protein content range and gas utilization. The ammonia requirements are estimated based on the reaction stoichiometry. No resource bottlenecks are identified in comparison to the current availability.

**TABLE 6:** Range of the share of global resources required to fulfill the minimum global human protein requirements, while accounting for 12% food waste.

		Low end	High end	
Protein content of methane SCP		80	50	%
Gas utilization		80	90	%
Methane SCP requirement		243	388	Megaton/year
Electricity capacity required		114	192	GW
Share of global electricity consumption		4.5	7.5	%
Natural gas required		524	1,062	bcm/year
Share of global natural gas production		12.5	25.3	%
Ammonia required		45		Megaton/year
Share of global ammonia production		26.3		%
Thermal energy requirements in terms of	Share of global natural gas production required	3.3	4.4	%
	Share of global coal production required	2.0	3.3	%

Even when accounting for fulfilling the entire global caloric requirements, the share of global natural gas that would have to be leveraged does not exceed 100%. At most, it would be 97% when assuming low gas utilization and use of natural gas for fulfilling the thermal requirements of the plants. The share of global electricity required in this case would be at most 25%. In comparison, the share of the global ammonia production capacity required would be 110% of the current values.

**FIGURE 3:**
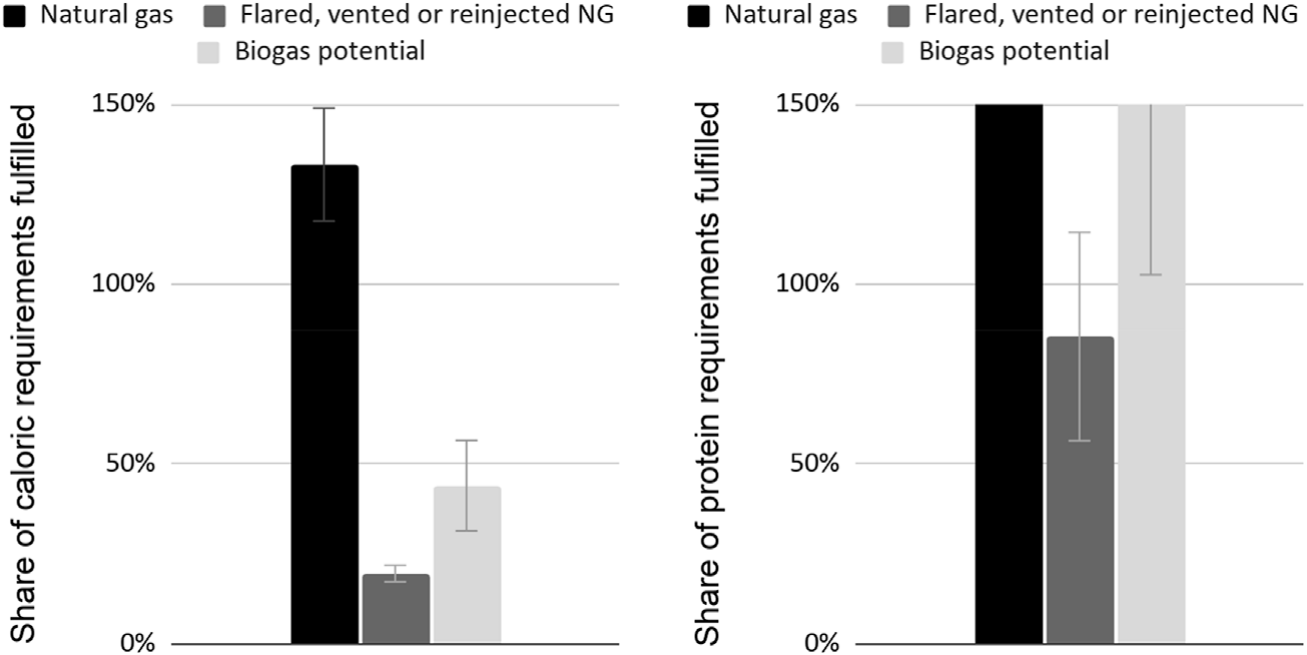
Share of the global caloric requirements that could be fulfilled by different potential methane sources (left) in comparison with the share of global protein requirements that could be fulfilled using the same sources (right).

The amounts of natural gas required to fulfill the global caloric and protein requirements *via* methane SCP are shown in Figure 3 in comparison to different available sources of methane. If the current production of flared, vented, and re-injected gas could be leveraged in its entirety for methane SCP production, it could cover 56%–100% of the methane needed to fulfill global protein requirements, depending on the protein content of the SCP, methane content of the gas, and gas utilization. The nitrogen contained in the current production of sewage and manure is estimated between 3–13 megaton/year (Matassa et al., 2020), equivalent to 8%–36% of the nitrogen requirements for fulfilling the global protein requirements. However, it is unclear whether this could be leveraged for SCP production during a GCFS.

Bacterial SCP production also requires a number of minerals for cell growth metabolism. These include calcium, iron, phosphorus, sulfur, sodium, chlorine, potassium, and magnesium, among others. Out of these, magnesium was the only one identified as a potential bottleneck to ramp-up SCP production (García Martínez et al., 2021b), but only when aiming to fulfill global caloric requirements.

### 3.3 Ramp-Up Speed Values

**FIGURE 4:**
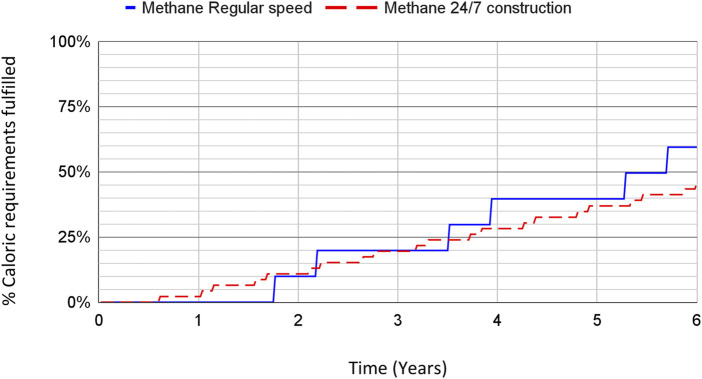
Expected ramp-up speed of methane SCP production in terms of the global caloric human requirements fulfilled over time. The results shown reflect the use of the budget of similar industries, including regular and fast construction speeds.

**FIGURE 5:**
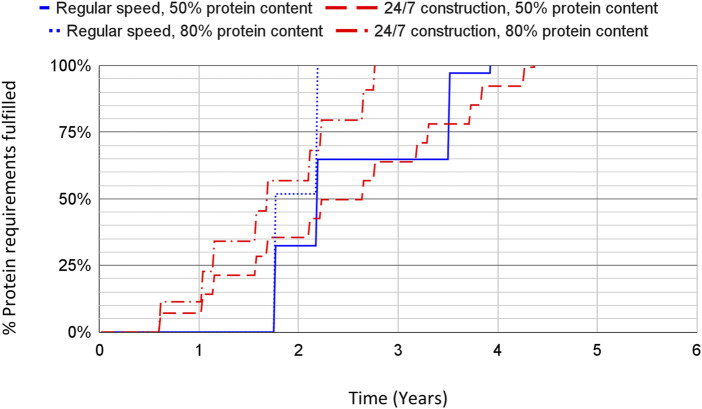
Expected ramp-up speed of methane SCP production in terms of the global protein human requirements fulfilled over time, for different values of the protein content of the SCP product. The results shown reflect the use of the budget of similar industries, including regular- and fast-construction speeds.

The construction time for a reference size plant is estimated at 87 weeks, and at 27 weeks when using 24/7 construction. The ramp-up speed for the scenario in which the global budget for chemical and other related industries can be effectively redirected to fast construction of methane SCP factories is shown in Figure 4 for the global caloric requirements and Figure 5 for the global protein requirements. For the fast construction scenario at the end of the first year, around 2% of the caloric requirements could be fulfilled, translating to 7%–11% of the protein requirements. The global protein requirements could potentially be covered in approximately 2.5–4.5 years. Note that the regular construction speed eventually overtakes the 24/7 construction due to its lower resource intensity, but takes much longer to start producing food which makes it worse in the advent of a GCFS.

For reference, if assuming unlimited capital and no bottlenecks, a capital cost of 6.1 trillion USD would be sufficient for building the amount of methane SCP factories required to fulfill the caloric requirements of humanity. This would take an amount of time equivalent to that of building one reference scale production plant at around 13 months to full production (see the second step in the curves from Figures 3, 4). Assuming slow construction, the capital cost would amount to 4.1 trillion USD, corresponding to a timeframe of 27 months until full production levels would be achieved.

Humans are unlikely to survive by relying on methane SCP as their sole food source. Aiming to fulfill all global human caloric requirements through SCP alone is unrealistic; however, methane SCP could potentially fulfill the protein requirements of the global population over the duration of the proposed sunlight reduction scenario, approximately equivalent to 19%–31% of the caloric requirements. This amount of protein could be provided through SCP production on a construction budget of 1.2–1.9 trillion USD, a range that assumes fast construction methods and varies depending on the protein content per unit of SCP product, at a fast construction budget of around 1.2–1.9 trillion USD depending on the protein content. The middle of the range of protein content, 65%, corresponds with the protein content expected from the Unibio SCP reference, assuming it has had nucleic acids removed, so it can be considered to constitute a “best estimate” of the ramp-up speed. Based on it, the protein requirements would be fulfilled in 3.5 years with the limited construction budget. A similar calculation based on a 9.8% fat content of the methane SCP (Silverman, 2020) and a minimum recommended fat intake equivalent to 15% of total energy intake (García Martínez et al., 2022) yields about 25% coverage of the global fat requirement when fulfilling the entire global protein requirement *via* methane SCP in this way. Other resilient foods could cover the rest of the nutritional requirements (Pham et al., 2022).

### 3.4 Food Price

**FIGURE 6:**
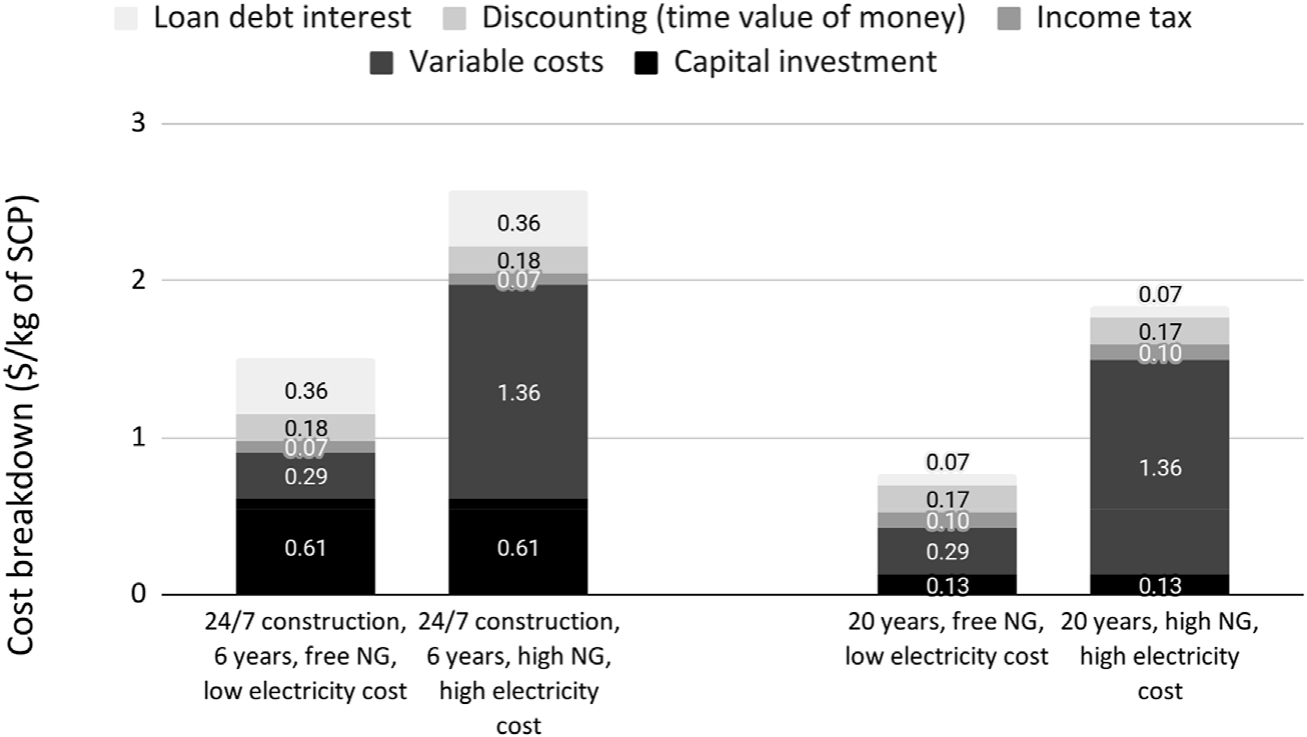
Breakdown of the contributions to the wholesale production cost incurred per unit of methane SCP produced.

The NPV analysis was performed to estimate the break-even cost of the methane SCP product for different scenarios. The expected cost of the SCP in the ASRS was estimated by limiting the plant life to 6 years and accounting for the additional cost of the 24/7 construction. For comparison, the product cost in regular conditions (20 years of lifetime and a regular construction cost) was also obtained. For each of the two scenarios, the product cost was calculated for a scenario of high-operating costs (high natural gas and electricity cost), and for an optimistic scenario using free natural gas (i.e., vented or flared) and low electricity cost, as lower and upper bounds for the cost. The results are shown in Figure 6.

A markup of 100% was applied to estimate the retail cost of the SCP product, accounting for the distribution and other additional costs (McCray, 2010). We refer to these values as retail cost instead of price due to the uncertain equilibrium of the market during a GCFS, which could alter the sale price. The result is shown in Table 7. The retail cost for fulfilling a person’s daily caloric requirements would be $1.19–2.03.

**TABLE 7:** Retail cost of methane SCP for different cost scenarios in U.S. dollars per kilogram of dry SCP product.

Scenario	GCFS conditions (6 years plant lifetime, 24/7 construction)		Regular conditions (20 years plant lifetime, regular construction)	
Energy and feedstock cost	Free NG, low electricity	High	Free NG, low electricity	High
Wholesale price (USD/kg)	$1.51	$2.58	$0.77	$1.84
Retail cost (USD/kg)	$3.02	$5.16	$1.54	$3.68

## 4 Discussion

The product retail cost for GCFS conditions does not significantly increase when comparing the scenarios of low- versus high-operating costs, the difference being US$3–5/kg dry. The difference in retail cost between regular and GCFS conditions is estimated at approximately $2/kg. In an ASRS, the sunlight levels could be reduced for longer than 6 years, but even with this longer delay before sunlight recovered to current levels, factories built later would still have fewer years of operation during which SCP products could command higher prices due to high demand. There would be lower demand for the SCP product after agricultural productivity recovers, although there could be some remaining opportunities for these plants to operate. The cost analysis is likely conservative for factories operating in the first year of the ASRS. At this price, it is expected that around 90% of the global population would be able to afford the SCP product for all calories with their current incomes (Denkenberger et al., 2019).

An important caveat regarding the cost estimations is that the financial assumptions used are common during business as usual, but the financial conditions of an ASRS are complex and outside of the scope of this work. There is considerable uncertainty; although governments gave interest-free loans during the COVID-19 pandemic, raising capital during a GCFS could conversely become far more difficult in the financial ecosystem. Further research is needed on market equilibrium during an ASRS for more precise price estimations of resilient foods.

As shown in Figure 3, it is conceivable that most or all of the methane required to fulfill the global protein requirements could be sourced exclusively from a combination of biogas and natural gas associated with oil which is currently being flared or re-injected. This means that methane SCP production may require redirecting little or no additional natural gas production capacity from current or future uses in order to fulfill this goal. Locations with an abundance of flared or vented natural gas should be prioritized for lower production costs. Additionally, these would not require redirecting the current natural gas production from its use as fuel, which would be a significant advantage during a nuclear winter. Using the natural gas feedstock as an energy source for fulfilling the thermal and electricity energy requirements of the plant could be more convenient than using other sources, but doing so would reduce the amount of feedstock available for further ramp-up. Additionally, further research on the availability of biogas during an ASRS would help provide more insights on the share of natural gas production capacity that would have to be redirected. Biogas produced from decaying plant biomass killed by an ASRS could be significant. Biogas could likely more efficiently be leveraged by co-cultivation of the methane-oxidizing bacteria with hydrogen-oxidizing bacteria (Kerckhof et al., 2021).

Methane SCP is generally faster to ramp-up compared to other industrial solutions for resilient food production in ASRS, such as new constructions of lignocellulosic sugar plants (Throup et al., 2022) or H_2_ SCP plants (García Martínez et al., 2021b). Other non-industrial, low-tech resilient food solutions such as tropical greenhouses (Alvarado et al., 2020) and seaweed farming in the ocean (Mill et al., 2019) are expected to scale up production faster. However, the high-protein content and quality of the methane SCP product far surpass those of the faster scaling solutions, making it valuable as a protein supplementation food during a GCFS. For this reason, a recommendation would be to limit the ramp-up of methane SCP to the production capacity required to fulfill global protein requirements at most, while the rest of the calories and nutrients are fulfilled by faster scaling resilient food solutions that the SCP nutritionally complements (Pham et al., 2022).

Regardless of the ramp-up speed of methane SCP, it would be a great advantage to have multiple factories built and operating before the strike of a GCFS. This would imply a head-start in terms of food production, equivalent to shifting the ramp-up curves upward. If the factories had originally been built for producing animal feed, adding a nucleic acid removal treatment would allow obtaining valuable human food early in the catastrophe. For each plant (of the reference size) available at the onset of an ASRS, an estimated maximum of 620,000 people could meet their entire caloric needs with the SCP product; an estimated maximum of 2.6 million people could meet their minimum protein requirements through the product. Some potential interventions to expedite the ramp-up of SCP technology that were previously identified apply for the case of methane SCP. A coordinated response plan for the materials and labor deployment to a collection of pre-approved sites would accelerate the initial response to the GCFS. A readily available, generalist methane SCP plant front-end engineering design package, whether produced by the industry or academia, could serve as a design basis for the new plants, similar to how Humbird et al., 2011 serves as a benchmark case study for the biochemical ethanol production process. In addition, once the sector has reached maturity, industry experts could create a guide on building and operating the plants, apart from sharing lessons on how to successfully reach and maintain production at scale (García Martínez et al., 2021b). Studies have estimated that effective work on the resilience to GCFS is highly cost-effective for the long-term (Denkenberger et al., 2022) as well as for the current generation (Denkenberger and Pearce, 2016; Denkenberger and Pearce, 2018a).

As shown in Figure 5, the protein content of the SCP product significantly influences the speed at which the global protein requirements can be fulfilled. If feasible, research on increasing the protein content would be of important use to this end, be it *via* optimization of operational parameters such as the methane to nitrogen ratio (Valverde-Pérez et al., 2020), use of thermophilic methanotrophs for reduced cooling requirements (El Abbadi et al., 2022), genetic modification of the microbes, or other means. These types of optimizations, if performed prior to a GCFS, would increase the potential of SCP as a resilient food.

The basis of the ramp-up model is the assumption that only the capital budget of chemical and other related industries (489 billion USD) could be leveraged for the construction of SCP factories, roughly accounting in this way for uncertainties in the availability of construction materials, construction of chemical equipment, and retraining of qualified labor. A previous order-of-magnitude estimate based on growth-rate values suggested that 100% of the global human caloric requirements could be fulfilled by methane SCP at around 6 months (Denkenberger and Pearce, 2015), in comparison to the 10 years estimated in Section 3.3. However, the current estimate is considered to be conservative. If the potential of the global construction industry to provide these resources could be effectively leveraged in its entirety, methane SCP could potentially fulfill the entire global caloric requirements of humanity in the time it would take to build a single wave of factories using 24/7 construction, or just over a year. This is because the annual construction expenditure is estimated at 12 trillion USD (de Best, 2021), surpassing the capital requirements for fast construction of methane SCP at 6.1 trillion USD for fulfilling caloric requirements.

Even with the current conservative estimate, there seem to be no significant impediments in securing the global protein supply using methane SCP within a nuclear winter period. Nonetheless, the time this would take is still longer than the 3–6 month period that global food reserves are expected to last (Denkenberger et al., 2019), and would require unprecedented international cooperation. More research is needed on the labor, equipment construction, and coordination constraints, so that the degree to which they would hinder the ramp up of methane SCP production (or production of other resilient foods for GCFS) is better understood. Similar to how a wide array of distributed production occurred to overcome supply shortages during the COVID-19 pandemic, it is expected that distributed production of SCP technologies would create greater resiliency in the food system. Similar to the calls for open hardware to prevent medical shortages (Chagas et al., 2020; Pearce, 2020b, 2020a), the development of open source scalable components of the small-scale SCP production systems is possible. Some of the units are amenable to additive manufacturing and are thus distributed open source production. There has already been substantial progress made on various heat exchangers (Arie et al., 2017; Denkenberger and Pearce, 2018b; Tiwari et al., 2019). In addition, there are open source desktop centrifuges that could be scaled for this application (WareJoncas et al., 2018; Sule et al., 2019). Additional work is needed to develop low-cost open sources and easily manufactured flash evaporators, spray dryers, cell homogenizers, gas compressors, and appropriate bioreactors with high gas utilization.

Low-tech alternatives for downstream processing in small scale SCP production could be settling trays in a refrigerator instead of a centrifuge and boiling the resulting concentrate instead of using a cell homogenizer and spray dryer. Small scale production could use air as the O_2_ source to obviate the need for air separation, at the cost of reduced gas utilization. Though small-scale production of methane SCP would be less efficient, it could have the feature of the waste heat heating the house (particularly advantageous in the case of nuclear winter). There would not be additional retailing costs in contrast to a large scale production. Safety issues, and the impact of fugitive methane emissions from small, less controlled “home” or “community” SCP production facilities would have to be assessed. We consider large, industrial SCP production plants more realistic than small-scale efforts, as the process requires proper control. Supplying strains and nutrients for local small-scale productions also seems hard to achieve.

Re-purposing the existing infrastructure for SCP production could expedite a GCFS response, and has already been studied for sugar production in the ASRS context (Throup et al., 2022). Similar to the methodology used in the sugar work, a unit-to-unit comparison of the methane SCP production process was made with several different industries, including breweries, instant coffee, milk powder, whey protein, washing powder, cheese powder, fertilizers, and biorefineries, showing some overlap. Whey protein factories already have units present for centrifugation, spray drying, packaging, and storage of the powdered product, but the global whey production capacity is insignificant compared to that required to fulfill the global protein requirements (∼1% of 0.24–0.39 Gt SCP/year required). On the other hand, the global production capacity of cheese powder and fertilizer factories together amount to a larger tonnage than would be required to fulfill the protein requirements *via* SCP production [0.093 and 0.317 Gt, respectively (FAO, 2017; GIR, 2020)]. If the drying process present in those factories could be leveraged in combination with the utilities, packing, power, steam, buildings, and facilities, saving upward of 40% of the total capital cost could potentially be obtained based on the Unibio reference plant (Jorgensen, 2011). The value is in accordance with a previously published analysis on repurposing paper mills to biorefineries, which similarly predicts a capital savings of 40%, mostly from leveraging existing buildings and service facilities (Martinkus and Wolcott, 2017). However, the very specific nature of the equipment required for SCP production could make it impracticable to repurpose existing units, particularly some of the costliest ones such as reactors, centrifuges, and spray dryers. Future research will study the potential of repurposing the existing infrastructure for SCP production in depth given the significance of expediting GCFS response.

This much needed future research can be viewed as a relatively high priority for GCFS mitigation and preparedness funding, but could also be viewed as a means of insurance for conventional methane infrastructure. As the science behind climate change becomes more granular, risks and liabilities for companies responsible for GHG emissions increase (Allen, 2003; Kunreuther and Michel-Kerjan, 2007; Faure and Peeters, 2011; Heidari and Pearce, 2016) and there is a mounting pressure to mitigate the climate liability risk. Although natural gas is less carbon intensive than coal, natural gas pipelines have recently been identified as high-priority targets for strategic lawsuits to act as GHG emission bottlenecks (Pascaris and Pearce, 2020). Natural gas companies could argue that their infrastructure could provide security during GCFS events that impact food systems as they make the transition to hydrogen carriers in a green economy if the future work outlined here to make SCP viable and easily scaled is funded and deployed globally.

## 5 Conclusion

The estimated capital cost for a methane SCP facility for human food production is $4,800/tpa when built using 24/7 construction to quickly produce protein-rich food during a global catastrophic food shock. The expected retail cost of the product would be in the range of $3–5/kg (dry), depending on the cost of the natural gas feedstock and electricity.

The ramp-up time of methane SCP is estimated at 2.5–4.5 years for fulfilling the protein requirements of the global population, within the duration of a nuclear winter scenario. No further scaling is recommended given the presence of faster scaling resilient foods. 7%–11% of the global protein requirements could be fulfilled at the end of the first year. Any facilities constructed prior to an abrupt sunlight reduction scenario would imply an important head start in food production capacity, given the sudden nature of the catastrophe and a limited window for adaptation.

No significant resource bottlenecks have been found regarding the inputs of methane SCP required to fulfill the global protein requirements. Potential bottlenecks regarding the availability of construction materials, construction of chemical equipment, and retraining of qualified labor were considered limiting. If these were not significant, protein requirements could potentially be fulfilled in little over a year. More research is needed on the degree to which these bottlenecks would affect SCP ramp-up speed.

Other important future research studies could include the re-purposing potential of existing facilities, economic conditions, and biogas potential for methane production during an abrupt sunlight reduction scenario, or the creation of open source designs for small scale production. Recommended interventions include stimulating SCP production during business as usual, creation of a coordinated response plan for materials, and the construction and development of open generalist SCP front-end engineering design packages and guides.

In conclusion, methane SCP has a significant potential to prevent global protein starvation during a global catastrophic food shock at a price affordable by most of the world’s population.

## Data Availability

The original contributions presented in the study are included in the article; further inquiries can be directed to the corresponding authors.

## Abbreviations

ASRS, abrupt sunlight reduction scenario; CAPEX, capital expenditure; CO_2_, carbon dioxide; GCFS, global catastrophic food shock; NG, natural gas; NPV, net present value; SCP, single cell protein.
